# The Role of Immune Checkpoints after Cellular Therapy

**DOI:** 10.3390/ijms21103650

**Published:** 2020-05-21

**Authors:** Friederike Schmitz, Dominik Wolf, Tobias A.W. Holderried

**Affiliations:** 1 Department of Hematology, Oncology and Rheumatology, University Hospital Bonn, 53127 Bonn, Germany; friederike.schmitz@ukbonn.de (F.S.); dominik.wolf@i-med.ac.at (D.W.); 2 UKIM 5, Hematology and Oncology, Medical University Innsbruck, 6020 Innsbruck, Austria

**Keywords:** cellular therapy, allogeneic stem cell transplantation, CAR T cell therapy, immune checkpoints, checkpoint inhibition, GvL, GvHD

## Abstract

Cellular therapies utilize the powerful force of the human immune system to target malignant cells. Allogeneic hematopoietic stem cell transplantation (allo-HCT) is the most established cellular therapy, but chimeric antigen receptor (CAR) T cell therapies have gained attention in recent years. While in allo-HCT an entirely novel allogeneic immune system facilitates a so-called Graft-versus-tumor, respectively, Graft-versus-leukemia (GvT/GvL) effect against high-risk hematologic malignancies, in CAR T cell therapies genetically modified autologous T cells specifically attack target molecules on malignant cells. These therapies have achieved high success rates, offering potential cures in otherwise detrimental diseases. However, relapse after cellular therapy remains a serious clinical obstacle. Checkpoint Inhibition (CI), which was recently designated as breakthrough in cancer treatment and consequently awarded with the Nobel prize in 2018, is a different way to increase anti-tumor immunity. Here, inhibitory immune checkpoints are blocked on immune cells in order to restore the immunological force against malignant diseases. Disease relapse after CAR T cell therapy or allo-HCT has been linked to up-regulation of immune checkpoints that render cancer cells resistant to the cell-mediated anti-cancer immune effects. Thus, enhancing immune cell function after cellular therapies using CI is an important treatment option that might re-activate the anti-cancer effect upon cell therapy. In this review, we will summarize current data on this topic with the focus on immune checkpoints after cellular therapy for malignant diseases and balance efficacy versus potential side effects.

## 1. Introduction

The human immune system is one of the most powerful weapons in the treatment armamentarium against cancer. Many different immune cell types play distinct roles in the concert of cellular anti-tumor immunity [1]. However, divers mechanisms evading this anti-tumor immune response have been recognized, including a weakened cellular immune system [2,3,4,5,6]. Cellular therapies have been established to overcome this hurdle. They either aim to replace the dysfunctional immune system with a new one from a healthy donor in case of allogeneic stem cell transplantation (allo-HCT), or to add highly specific genetically modified autologous T cells in CAR T cell therapy. Despite these potent treatment options, disease relapse remains a major concern and further options for increasing anti-tumor immunity are under intense investigation. Inhibitory immune checkpoints play a pivotal role in limiting the hyperactivation of the immune response. In the setting of malignant disease, these mechanisms limit an effective anti-tumor immune response. Blocking antibodies targeting inhibitory immune checkpoints have been successfully implemented into the standard treatment algorithms of various malignancies for almost a decade. However, the effect of immune checkpoints in relapse after cellular therapies, such as allogeneic stem cell transplantation (allo-HCT) and CAR T cell therapy has yet to be fully explored. Additionally, the role of checkpoint inhibition (CI) after cell therapies is under current investigation. This review aims to elucidate the role of immune checkpoints after cellular therapies, as well as ways to modulate the immune function after cell therapy with CI for potential prevention or treatment of relapse. Next to pre-clinical and early clinical data elucidating the relationship between immune checkpoints and relapse after cell therapy, analysis of efficacy and responses and the view on toxicities of CI after cellular therapy are crucial points of this review.

### 1.1. Allogeneic Hematopoietic Stem Cell Transplantation (Allo-HCT)

Already in the late 1950s, Thomas et al. [7] conducted the oldest successfully realized cell therapy by performing the first allogeneic hematopoietic stem cell transplantation (allo-HCT). Initially, allo-HCT was considered to be a therapeutic approach for alleviating the toxic side-effects of high dosage radiation and chemotherapy by replacing the patient’s bone marrow with a new hematopoietic system from a healthy donor [7]. Since then, allo-HCT has become a potentially curative treatment option for various high-risk hematological malignancies as well as benign bone marrow diseases. Eventually, it became more apparent that the efficacy of allo-HCT in malignant diseases was owed to immunologically active cells of the donor against cancer cells of the host, the so-called Graft-versus-leukemia (GvL) effect [8]. However, already early observations affirmed that immunologically active donor cells also cause one of the major and potentially lethal complications of allo-HCT, the Graft-versus-Host Disease (GvHD) [9]. While the outcome after allo-HCT has been greatly improved, especially regarding the prevention and treatment of GvHD and infections, disease relapse is still a major complication after allo-HCT. While the GvL effect is considered to be mainly responsible for the cure of the underlying disease, the loss of this donor-mediated immunological anti-tumor effect is considered to be a major factor for relapse after transplant [10,11]. The restoration of the GvL effect while minimizing the complications of GvHD is a main challenge in relapse after allo-HCT [12]. Donor lymphocyte infusions (DLI) [13] are one possibility for restoring the GvL effect by boosting the host’s immune system. However, with only limited success with current treatment options in relapsed patients after allo-HCT ways to restore the GvL effect are urgently needed. One tempting approach to achieve this goal is to combine allo-HCT with CI.

### 1.2. Chimeric Antigen Receptor T Cells (Cars)

With the recent implementation of CARs, entirely novel cellular treatment options in various malignancies have arisen. CARs are autologous genetically engineered T cells expressing a chimeric antigen receptor that is specific for a tumor-associated antigen. CARs consist of an extracellular single chain variable fragment (scFv), a transmembrane domain, and an intracellular signaling domain. The CAR technology was first developed in the 1980s and further modified before entering clinical practice [14,15,16,17,18,19,20,21,22]. First generation CARs were called “T-bodies”, which were solely built of a scFv with the CD3ζ intracellular signaling domain to induce T cell activation. Due to initially limited antitumor activity, one (2nd generation) or two (3rd generation) costimulatory molecule domains, respectively (e.g., CD28, 4-1BB (CD137) or OX-40), were added to the CD3ζ intracellular signaling domain. These costimulatory domains enhance activation, function and persistence stimulating their proliferation and cytokine release. Second generation CD19-CARs have been approved by the Food and Drug Administration (FDA) in 2017 and by the European Medicines Agency (EMA) in 2018 for patients with relapsed/refractory diffuse large B cell lymphoma (DLBCL), acute lymphatic leukemia (ALL < 25 years), and primary mediastinal B cell lymphoma (PMBCL), respectively. Significant side effects of CAR T cell therapy are cytokine release syndrome (CRS) and immune cell associated neurotoxicity syndrome (ICANS). While these side effects can have severe manifestations sometimes requiring intensive care treatment, they are mostly well manageable and temporary [23,24]. Long-term toxicities are rare, but the short follow-up time until now must be considered. However, disease relapse is still a major issue after CAR T cell therapy [25]. Antigen loss, exhaustion of CAR T cells, and the expression of immune checkpoints have been linked to relapse [26]. Therefore, a combination of CARs with CI to enhance their anti-tumor efficacy in expectation of higher cure rates is a feasible option.

### 1.3. Immune Checkpoint Inhibitors (CI)

The inhibition of immune checkpoints, such as programmed death-1 (PD-1) or cytotoxic T-lymphocyte-associated protein-4 (CTLA-4), have led to a breakthrough in cancer treatment by releasing the break on cellular anti-tumor immunity [3,27,28,29,30]. Checkpoint inhibition has evolved to be a well-established and very successful treatment option in various solid tumors, resulting in improved overall survival and even long-term tumor control in some cases [31,32,33,34]. CTLA-4 and PD-1 are central immune checkpoints that negatively regulate T cell immune function [29,35,36,37].

In 1996, James P. Allison et al. provided first evidence that anti-CTLA-4 inhibitory therapy improves anti-tumor immune responses in a pre-clinical murine cancer model [5]. Tasuku Honjo and colleagues showed that the engagement of PD-1 and its ligand PD-L1 (programmed death- ligand 1) on tumor cells lead to a potent inhibition of T cell receptor-mediated lymphocyte proliferation and cytokine secretion. This interaction could be reversed by application of anti-PD-L1 monoclonal-antibody (mAb), providing a promising treatment strategy for specific tumor immunotherapy [3]. The seminal findings of both researchers were awarded with the Nobel Prize for medicine in 2018 as a consequence of the ground-breaking nature of these findings for today’s cancer medicine.

Since ipilimumab’s FDA approval in 2011 for metastatic melanoma, five additional CI, including PD-1 and PD-L1 inhibitors (nivolumab, pembrolizumab, atezolizumab, avelumab, and durvalumab), were approved for numerous advanced solid malignancies and relapsed Hodgkin lymphoma. Alternative novel immune checkpoints in the tumor microenvironment have been detected as potential targets (e.g., T-cell immunoglobulin mucin-3 (TIM-3)/galectin-9, lymphocyte-activation gene 3 (LAG-3) or T cell immunoglobulin and ITIM domains (TIGIT)) in order to enhance the benefit from CI especially in patients with resistance to PD-1/PD-L1/CTLA-4 mAbs. Immune-related toxicities can, in some cases, be life-threatening, but are mostly well manageable when patients are properly monitored and early intervention is initiated [38]. While combinations of CI with other agents, such as conventional chemotherapeutics, have already been approved in some diseases, the role of CI after cellular therapy is still under investigation and it will be discussed throughout this review.

## 2. Immune Checkpoints and Checkpoint Inhibition after Allo-HCT

Multiple factors facilitating the loss of the GvL effect and, consequently, allowing disease relapse after allo-HCT have been described [39,40]. One reason for the lack or reduction of allo-immune T cell function might be due to the exhaustion of the T cells [41]. An exhausted T cell phenotype is associated with increased expression of inhibitory checkpoints (PD-1, TIM-3, and others) [42]. Several studies have been published analyzing the expression of inhibitory checkpoints in the context of disease relapse after allo-HCT. During the early post-transplantation phase, PD-1 was shown to be ubiquitously overexpressed on T cells, but interestingly without being a reliable predictive marker of potential disease relapse [43]. Hutten and colleagues found that high co-expression of PD-1, TIGIT, and killer cell lectin-like receptor subfamily member 1 (KLRG-1) on minor histocompatibility antigen (MiHA)-reactive CD8^+^ T cells correlates with disease relapse after allo-HCT [44]. In comparison to relapse-free patients after HLA-matched allo-HCT, a higher proportion of CD8^+^ T cells that accumulate in the bone marrow and express inhibitory molecules, like CTLA-4, PD-1, and TIM-3, were detected in patients with relapse. Curiously, this was not the case in HLA-haploidentical transplanted patients, possibly due to a higher degree of HLA-mismatch leading to an aggravated pro-inflammatory milieu in these patients [45]. Additionally, these T cells showed impaired immune function and an increase in the expression of CD80, CD86, PD-L1, and Galectin-9 was observed on leukemic blasts [45]. In-depth analyses of the immune signature of leukemic blasts during relapse after allo-HCT confirmed these findings and revealed, amongst others, an up-regulation of inhibitory checkpoints driving leukemia escape [46]. Further phenotypical and functional analyses of patient cells also display an important role of the PD-1/PD-L1 interaction in relapse after allo-HCT [47]. Kong et al. identified circulating CD8^+^ PD-1^hi^TIM-3^+^cells with reduced production of IL-2, TNF-α, and IFN-γ. These cells could already be identified after allo-HCT, but before diagnosis of overt leukemia relapse and thereby might possibly serve as a screening tool [48]. Elevated PD-1 expression on CD4+ T cells early after allo-HCT also correlates with increased mortality [49]. In addition to T cells, natural killer (NK) cells play a fundamental role in providing the GvL effect after allo-HCT [50]. Hattori et al. investigated the role of TIGIT in bone marrow samples after allo-HCT, which was—when highly expressed—associated with a decreased incidence of aGvHD, while overall survival (OS) and progression free survival (PFS) were poor [51].

These findings fueled the hypothesis that the inhibition of inhibitory immune checkpoints might be feasible in the prevention and treatment of disease relapse after allo-HCT. Preclinical murine studies showed that PD-1 blockade after allo-HCT can refuel the GvL effect [52,53,54]. In a murine GvHD model, the up-regulation of PD-1 on dysfunctional T cells during GvHD correlated with a loss of the GvL effect, while PD-L1 blockade was able to restore it [52]. The GvL effect of adoptively transferred genetically modified T cells expressing a T cell receptor against leukemia-associated antigen could also be enhanced by PD-L1 blockade in a murine model [53]. Additionally, it was shown that the PD-1/PD-L1 pathway engages in the compartmentalization of cytotoxic T cells in different tissue environments after allo-HCT, leading to tumor escape. The restoration of tumor control could be achieved by the application of CI [52,54].

In line with these results, early case reports regarding PD-1 blockade in patients with Hodgkin’s Disease (HD) that had relapsed after allo-HCT were published [55,56,57,58,59,60,61]. In those reports, all of the patients responded and none of them suffered from the induction of acute GvHD (aGvHD). Singh and colleagues firstly reported the induction of severe and eventually fatal skin and liver GvHD in a young patient with HD evolving one week after first pembrolizumab dose [62]. Haverkos et al. [63] conducted a multicenter retrospective study with 31 lymphoma patients (29 HD, one transformed follicular lymphoma (FL) and one with FL+ HD), who were treated with anti-PD-1 mAb after relapse following allo-HCT to better characterize the risks and benefits of PD-1 blockade after allo-HCT. Twenty-eight of those were treated with nivolumab and three with pembrolizumab. The overall response rate (ORR) was 77%, median PFS 591 days, and median OS was not reached because 21 of 31 patients were still alive at study termination at a median follow-up of 400 days. However, 17 patients developed GvHD after anti-PD-1 treatment, including eight patients with severe GvHD (six aGvHD and two cGvHD). In the same year (2017), Herbaux et al. published a retrospective analysis of nivolumab mono-therapy in 20 relapsed HD patients after allo-HCT, which again supported the efficacy of nivolumab [64]. ORR was 95% translating into a one-year-PFS of 58.2% and an OS of 78.7% after a median follow-up or 370 days. Six patients (30%), though, developed aGvHD after nivolumab initiation, of which five were reported as severe GvHD. There is only limited data regarding efficacy in the post-transplantation setting with CI in relapsed diseases other than HD. In a case of relapsed anaplastic large cell lymphoma after allo-HCT, low-dose pembrolizumab resulted in CR with tolerable side effects (liver GvHD) [65]. Albring et al. [66] published a case study of three patients with relapsed acute myeloid leukemia (AML) after allo-HCT, who received post-relapse treatment with nivolumab. Two patients responded and side effects, namely GvHD, could be well controlled. In a retrospective German multi-center study, 21 patients were examined, who had received off-label therapy with CI (nivolumab, ipilimumab, or the combination of both mAbs) alone or in combination with DLI for treatment of disease relapse after allo-HCT, (*n* = 15 after 1st; *n* = 5 after 2nd, and *n* = 1 after 3rd) [67]. Twelve patients suffered from relapsed AML or myelodysplastic syndrome (MDS), two from ALL, five from Non-Hodgkin-Lymphoma (NHL) and two from myelofibrosis (MF). ORR was 43% with three complete remissions (CR) and six partial remissions (PR). One patient had stable disease (SD) and 10 patients progressive disease (PD). ORR was 40% in patients receiving nivolumab, 80% when nivolumab was combined with DLI, and 20% in patients receiving ipilimumab. The development of aGvHD III-IV or moderate/severe cGvHD was seen in 29% of the patients. Especially patients receiving the combination of CI with DLI were at very high risk of GvHD development. Further immune-related toxicities were rare. When compared to ipilimumab, Davids and colleagues observed in a phase 1/1b study with nivolumab more severe GvHD and immune-related adverse events (irAEs), even when the lowest dose (0.5 mg/kg) was applied (median time 21 months after allo-HCT). Furthermore, shorter time from allo-HCT until application of CI was significantly associated with a higher risk of development of GvHD [68].

Kline et al. [69] examine pembrolizumab in a prospective, still recruiting clinical trial for the treatment of relapsed disease following allo-HCT (NCT02981914). In an early report, they presented eight patients with AML and three with lymphoma. Patients with AML showed discrete response to pembrolizumab (2 SD, 2 PD). irAEs were observed in 63% (any grade), which were well manageable. The first clinical trial using CTLA-4 blockade after allo-HCT (ipilimumab was administered at doses up to 3 mg/kg) demonstrated an acceptable safety profile [70]. Notably, the response to ipilimumab for the treatment of relapse after allogeneic transplantation is dose-dependent [71], as no objective responses were seen at a dose of 3 mg per kilogram body weight, whereas the best responses were seen among 22 included patients receiving 10 mg/kg of ipilimumab (7 CR/PR, 6 SD), including three patients with leukemia cutis. After 27 months median follow-up, OS and PFS were 54% and 32%, respectively. GvHD, which was steroid-sensitive, appeared in 14%. However, severe irAEs, of which one was fatal, were observed in six patients [71].

Additionally, the combinatory use of lenalidomide and ipilimumab after allo-HCT has shown good tumor control and significant increase of ICOS^+^ CD4^+^ FoxP3^−^ T cells, indicating a synergistic effect of these two agents. ORR was good (70%) and no severe irAEs or GvHD were induced [72]. Table 1 summarizes relevant studies regarding CI after allo-HCT. In further currently ongoing clinical trials, mono or dual CI therapy with PD-1 and CTLA-4 inhibition after allo-HCT in high risk relapsed/refractory (r/r) AML or MDS, but also the combination of one checkpoint inhibitor with hypomethylating agents after allo-HCT are currently being evaluated and the results are eagerly awaited.

## 3. Immune Checkpoints and Checkpoint Inhibition after Cars

The recognition and interaction of CAR T cells with the corresponding target cell leads to their activation and proliferation, to the recruitment of further immune cells, and to the release of pro-inflammatory cytokines, and thereby eliminates tumor cells. Besides the great antitumor effect induced by CAR T cells, unneglectable significant relapse rates still remain an unresolved clinical problem [18,73,74,75,76,77]. Several mechanisms of failure of CAR T cell therapy have been uncovered, including poor CAR T cell persistence, low intrinsic CAR T cell fitness, antigen loss and escape, trogocytosis, as well as an inhibitory tumor microenvironment [25,26,78,79,80,81]. The assessment of immune checkpoints after CAR T cell therapy revealed an up-regulation of PD-1 that was seen on CD4^+^ and CD8^+^ anti-CD19-CAR T cells after treatment and PD-1 expression was higher on CAR-T cells than on non-CAR T cells [82,83,84]. Gene signature analyses of patients from the ZUMA-1 trial additionally showed an increase in gene expression of PD-1, LAG-3, and CTLA-4 after CD19-specific CAR T cell treatment [85]. In melanoma patients that were treated with anti-GD2 CAR T cells the CAR T cells showed an up-regulation of PD-1 and PD-L1, which was associated with a limited persistence of the CAR T cells [86]. In the tumor microenvironment of glioblastoma, patients up-regulation of PD-L1 in the microenvironment was seen after receiving anti-epidermal growth factor receptor (EGFRvIII) CAR T cell treatment and associated with incomplete responses [87]. In murine studies, the up-regulation of PD-1, LAG-3, TIM-3, and 2B4 was associated with impaired anti-mesothelin CAR T cell function and the inhibition of PD-L1 could restore CAR T cell function in a xenograft mouse model [88]. The extracellular domain of PD-1 was fused to intracellular co-stimulatory domains to resist suppression by PD-L1 (e.g., CD28). This chimera led to enhanced PD-L1 dependent cytotoxic T cell stimulation with increased cytokine secretion [89]. In adoptive transfer studies in Her-2 transgenic recipient mice, it was shown that the combination of Her-2 targeted CAR T cell therapy with anti-PD-1 mAb significantly reduced tumor mass and prolonged survival of the tumor bearing mice. Combinatory use of gene-modified Her-2 T cells with anti-PD-1 mAb was well tolerated without any signs of autoimmunity in recipient mice [90]. The triple-downregulation of PD-1, TIM-3, and LAG-3 in anti-Her2 CAR T cells using short hairpin RNA cluster resulted in enhanced tumor control in mice [91]. CRISPR/Cas9 (clustered regularly interspaced short palindromic re-peats associated with Cas9 endonuclease)- based gene editing was used to knockout PD-1 on primary T cells, leading to enhanced cytotoxicity [92]. Combined CRISPR/Cas9- based gene editing of anti-CD19 CAR T cells with mediated disruption of PD-1 resulted in augmented killing efficacy of the CRISPR/Cas9-edited CAR T cells in vitro and potently cleared PD-L1^+^ tumor xenografts in vivo [93]. Approaches with CRISPR/Cas9 modulated allogeneic CAR T cells deficient in TCR and HLA class I, as well as PD-1, showed potent in vivo anti-tumor efficacy with reduced alloreactivity not causing GvHD [94,95]. Anti-CAIX CAR T cells that secrete PD-L1 antibodies in murine renal cell carcinoma [96] or anti-CD19 CAR T cells secreting PD-1-blocking single-chain variable fragments (scFv) [97] showed improved anti-tumor activity of CAR T cells that was superior to conventional CAR-T cells in vivo. The treatment with anti-PD-1 scFv-producing CAR-T cells appeared to have an increased concentration of PD-1 scFv in tumor tissue, but not in sera [98]. These results suggest a possibility to decrease adverse events that are caused by systemically applied PD-1 blockade. In xenograft AML models, a significant up-regulation of TIM-3 on CAR T cells was shown in relapsed mice in comparison to CAR T cells that were isolated from mice in remission after CAR T cell therapy [99]. Refueling the immunological response of the CAR adding PD-1 or TIM-3 blocking antibodies to the same mouse model showed improved response rates. Interestingly, this synergistic effect even further increased in the presence of both checkpoint inhibitors combined [99].

With these and other encouraging preclinical results, several initial case series combining checkpoint inhibition with CAR T cell therapy were published. One report of fourteen heavily pretreated patients with r/r B-cell ALL and B lymphoblastic lymphoma with early CAR T cell loss or insufficient response to anti-CD19 CAR T cell therapy received PD-1 blockade no sooner than fourteen days after CAR T cell infusion [100]. At least partial responses were reported in the majority of the patients without unexpected or fatal toxicities. In other cases of pembrolizumab (2 mg/kg) on day 26 [101] or nivolumab (3 mg/kg) treatment on day 11 [102] for progressive DLBCL after anti-CD19 CAR T cell therapy rapid responses were seen and further assessment showed a drastically increased CAR T cell number after PD-1 blockade when compared to patients not receiving CI. A retrospective study analyzed eleven patients with r/r lymphoma who received nivolumab (3 mg/kg) on day 3 after anti-CD19 CAR T cell infusion [103]. ORR was 81% and, therefore, not superior to patients that received anti-CD19 CAR T cell treatment alone. However, due to differing patient characteristics a direct comparison is not possible. Importantly, CI related toxicities in combination with CAR T cell therapy were again well manageable [103]. In an initial report of the phase 1b multi-center open-label PORTIA trial (NCT03630159), four patients with refractory DLBCL, receiving pembrolizumab on day 21 after anti-CD19 CAR T cells have shown, so far, no irAEs nor severe CRS/ICANS [104]. Early results of the phase 1 of ZUMA-6 trial (NCT02926833), in which 12 patients with r/r DLBCL received anti-CD19 CAR T cells followed by 4 infusions of atezolizumab, no worsening of CAR T cell related adverse events was noted following CI [105]. Encouraging OR of 90% as well as an increased CAR T cell expansion and persistence as compared to anti-CD19 CAR T cell therapy alone support phase 2 of ZUMA-6 [105]. Another approach to target tumor antigens in addition to disrupting the PD-1/PD-L1 interaction is the use of bicistronic CAR T cells (AUTO3) targeting the two antigens CD19 and CD22, followed by anti-PD-1 consolidation courses (NCT03287817) in patients with r/r DLBCL. Early data have shown early efficacy with the lowest CARs dose (50 × 10^6^ cells) and pembrolizumab 200 mg (given every three weeks) with an ORR of 57% and modest side effects [106].

In a phase I clinical trial (NCT03399448), CRISPR/Cas9 edited T cells with triple gene depletion including the PD-1 locus were infused after lymphodepleting chemotherapy into three patients with advanced refractory cancer (multiple myeloma, *n* = 2; liposarcoma, *n* = 1). The overall tolerance was good, with only mild toxicity and without the appearance of CRS. The best clinical responses were stable disease in the two myeloma patients, but eventually all three patients showed progress [107].

A growing number of CARs have been designed to target solid tumors, e.g., directed against EGFRvIII in glioblastoma showing prolonged CAR T cell persistence and the improvement of anti-glioma-activity with PD-1 knockout in a xenograft mouse model [108]. The combinatory use of EGFRvIII-CAR and pembrolizumab in patients with newly diagnosed 0-6-Methylguanine DNA-methyltransferase (MGMT)–unmethylated glioblastoma is currently being evaluated in a clinical trial (NCT03726515).

Encouraging synergistic effects have been described in early reports with the limited clinical data of treatment strategies combining CI with CAR T cells available (see Table 2). These strongly support further clinical trials utilizing immune checkpoints to optimize CAR T cell therapies. Table 3 illustrates the current overview with ongoing studies. The overall toxicity with this approach was not severely enhanced in the available, yet small, studies. However, special attention needs to be granted to the occurrence of autoimmune-related adverse events also in this setting [109].

## 4. Conclusions

**Figure 1:**
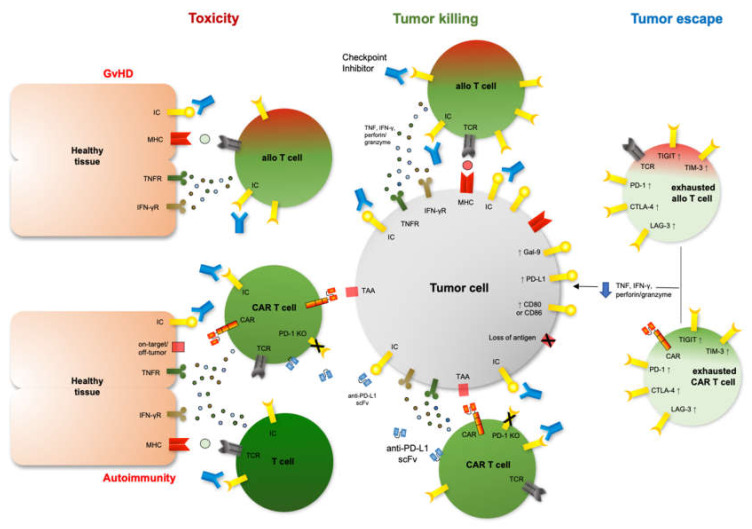
Mechanisms of tumor escape, restored anti-tumor immunity after blocked signaling of inhibitory immune checkpoints as well as therapy-mediated toxicity. Right: Up-regulation of inhibitory immune checkpoints (IC) like programmed death-1 (PD-1), cytotoxic T-lymphocyte-associated protein-4 (CTLA-4), T-cell immunoglobulin mucin-3 (TIM-3), lymphocyte-activation gene 3 (LAG-3) or T-cell immunoglobulin and ITIM domains (TIGIT) on exhausted T cells after cell therapy leads to impaired tumor recognition and killing. Center: Therapeutic strategies modifying inhibitory ICs, i.e., checkpoint inhibitors or CAR T cells expressing anti-PD-L1 single chain variable fragments (scFv) or harboring a PD-1 knockout (KO), can restore anti-tumor immunity after cellular therapy. Left: Increased immune function after blocked inhibitory immune checkpoints may amplify undesired toxic side effects on healthy tissue after cell therapy such as Graft-versus-Host-Disease (GvHD), immune-related adverse events (irAEs) or increased on-target/off-tumor activity.

Immunotherapy of cancer has revolutionized the treatment landscape in oncology. Strategies targeting immune checkpoints are also entering late clinical testing in hematology (i.e., TIM-3 mAb in MDS/AML) or are already approved (PD-1 blocker in cHL). In addition, allo-HCT and CAR T cells are established cellular therapies in hematology; however, both of them are also characterized by high relapse rates. One cause of relapse after cell therapy is an impaired T cell function accompanied with increased expression of inhibitory immune checkpoints. The currently available data shows the up-regulation of inhibitory immune checkpoints after cell therapy. Higher levels of immune checkpoints, such as PD-1/PD-L1, CTLA-4, TIM-3, LAG-3, or TIGIT, appear to be associated with increased relapse rates and reduced survival. Thus, it is tempting to speculate that the combination of cellular therapy with immune checkpoint blockade is a rational approach for improving outcome after cellular therapy. Preclinical data supported this hypothesis, which led to the individual clinical application and initiation of clinical studies of CI after allo-HCT and CAR T cell therapy. First results with PD-1 or/and CTLA-4 blockade indicate best responses in relapsed HD, but also other hematological malignancies, such as acute leukemia and NHL, show adequate responses to CI after allo-HCT. On the one hand, immune-related adverse events after CI in the setting of allo-HCT appear mostly well manageable, but GvHD induction after CI is often lethal or confers high morbidity burden, even though the underlying disease may be pushed back. On the other hand, a combination of CI with CAR T cell therapy appears mostly safe, while auto-immune related toxicities must be closely considered. Early reports fuel the hope of synergistic effects with this combinatory strategy. Additionally, genetically modified CRISPR/Cas9-based CAR T cells with specific checkpoint-knock-outs show good anti-tumor-efficacy without bearing uncontrollable toxicity. Figure 1 provides an overview of the mechanism of tumor escape, restored anti-tumor immunity after blocked signaling of inhibitory immune checkpoints as well as therapy-mediated toxicity.

To completely answer the question which immune checkpoint in cellular therapy is dominant with respect to resistance evolvement and, thus, representing the most appropriate therapy target warrants further investigations. This might not only be the PD-1/PD-L1 axis or CTLA-4, but also involves other candidates, such as TIM-3/galectin-9, TIGIT, or LAG-3. When considering a combination of checkpoint inhibition with cellular therapies, many aspects regarding their optimal use have to be taken into account to minimize the toxic side effects while guaranteeing optimal efficacy. Especially the identification of the appropriate patient population as well as optimal dosing, sequencing and therapy duration schedules have to be established in future studies, as they are inevitable to ensure the safety and efficacy of this approach.

## Figures and Tables

**Table 1:** Overview of relevant studies targeting immune checkpoints after allogeneic hematopoietic stem cell transplantation.

n	Disease	Characteristics	Intervention	Response	irAEs (Grade)	GvHD (Grade)	Ref.
31	r/r HL/FL	retrospective multi-center study HL, *n* = 29; transformed FL, *n* = 1; FL + HL, *n* = 1 85% received ≥ 1 salvage therapy post allo-HCT before anti-PD-1 mAb prior GvHD NOS (19/31)	nivolumab (q2w, 3 mg/kg) *n* = 28 pembrolizumab (q3w, 200 mg) *n* = 3 first application 26 mo. after allo-HCT (median)	ORR/CR/PR/SD/PD: 77/50/27/10/13% OS: N/A PFS: 591 days	N/A	aGvHD (N/A, *n* = 6), overlap (N/A *n* = 4), cGvHD (N/A, *n* = 7) In 16/17 GvHD onset after 1–2 doses of CI 8 GvHD related deaths	[63]
20	r/r HL	retrospective multi-center study HL, *n* = 20 65% received ≥ 1 salvage therapy post allo-HCT prior aGvHD (10/20), cGvHD (3/20)	nivolumab (q2w, 3 mg/kg) first application 23 mo. after allo-HCT (median)	ORR/CR/PR/PD: 95/42/52/5% OS/PFS: 79/58%	cerebellar ataxia (II, *n* = 1) hepatitis (II, *n* = 7)	aGVHD (I, *n* = 1; III, *n* = 3; IV, *n* = 2) In 6/20 GvHD onset after 1 dose of CI 2 GvHD related deaths	[64]
21	r/r HM	retrospective multi-center study AML/MDS, *n* = 12; ALL, *n* = 2; NHL, *n* = 5; MF, *n* = 2 relapse after 1st allo-HCT, *n* = 15; 2nd, *n* = 5; 3rd, *n* = 1 prior aGvHD (13/21), cGvHD (7/21)	nivolumab (0.5 mg/kg, 3 mg/kg, 40 mg or 200 mg absolute), *n* = 5 nivolumab + DLI (0.5 mg/kg, 1 mg/kg, or 40 mg absolute), *n* = 5 nivo + ipilimumab (both 3 mg/kg), *n* = 1 ipilimumab (3 mg/kg, or 10 mg/kg), *n* = 10 first application 4.5 mo. after allo-HCT (median)	ORR/CR/PR/SD/PD: 43/14/29/4/48% ORR (nivolumab): 40% ORR (nivo + DLI): 80% ORR (ipilimumuab): 20% OS: 79 days PFS: N/A	N/A	10/21 (any grade) 6/21 aGvHD III/IV or moderate/severe cGvHD; 5/5 GvHD in patients with nivo + DLI In 6/20 GvHD onset after 1 dose of CI 4 GvHD related deaths	[67]
11	r/r AML r/r (N)HL	phase I prospective single-center study AML/MDS, *n* = 8, lymphoma (DLBCL, HL), *n* = 3 no prior aGvHD > I or cGvHD	pembrolizumab (200 mg q3w) first application after allo-HCT N/A	ORR/CR/PR/SD/PD: 28/28/0/28/43% OS/PFS: N/A	7/11 (any grade) pneumonitis (III-IV, *n* = 2), hyperthyrodism (III, *n* = 1), rash (II, *n* = 1) onset after 1-2 cycles of CI	none	[69]
29	r/r hematologic and solid malignancies	phase I multi-center study HL, *n* = 14; Myeloma, *n* = 6; AML, *n* = 2; CML, *n* = 2; CLL, *n* = 2; NHL, *n* = 1; breast cancer, *n* = 1; renal cell cancer, *n* = 1 no ongoing GvHD, no prior III/IV GvHD	ipilimumab (+ DLI if progressive after CI) single dose 0.1 mg/kg, *n* = 4 single dose 0.33 mg/kg, *n* = 3 single dose 0.66 mg/kg, *n* = 4 single dose 1 mg/kg, *n* = 3 single dose 3 mg/kg, *n* = 15 application after allo-HCT N/A	CR: 2/29 (cHL) PR: 1/29 (NHL) OS/PFS: N/A	polyarthropathy (III, *n* = 1); hyperthyrodism (I-II, *n* = 1); dyspnea on exertion (N/A); pneumonitis (IV, *n* = 1)	no grade III-IV time of GvHD onset after CI N/A GvHD related deaths N/A	[70]
28	r/r HM	phase I/Ib multicenter study AML, *n* = 12; HL, *n* = 7; NHL, *n* = 4; MDS, *n* = 2; MM, *n* = 1; MPN, *n* = 1; ALL, *n* = 1 72% received ≥ 1 salvage therapy post allo-HCT before anti-PD-1 mAb no ongoing GvHD, no prior III/IV aGvHD	ipilimumab (q3w, 4 courses) 3 mg/kg; *n* = 6 3 mg escalated to 10 mg/kg; *n* = 7 10 mg/kg; *n* = 15 first application 56 mo. after allo-HCT (median)	ORR/CR/PR/SD/PD 23/9/27/41% (10 mg/kg) no response with 3 mg/kg OS/PFS: N/A	6/28 (any grade) pneumonitis (II-IV, *n* = 3) colitis (III, *n* = 1) ITP (II, *n* = 1) diarrhea (II, *n* = 1) death (*n* = 1)	with 10 mg/kg ipilimumab aGvHD gut (N/A, *n* = 1) cGvHD liver (N/A, *n* = 3) time of GvHD onset after CI N/A GvHD related deaths N/A	[71]
28	r/r HM	phase I/Ib multi-center study AML, *n* = 11; MDS *n* = 7; HL, *n* = 5; NHL, *n* = 3; MPN, *n* = 1; CLL, *n* = 1 64% received ≥ 1 salvage therapy post allo-HCT	nivolumab (q2w) 1 mg/kg (initial dose), *n* = 6 0.5 mg/kg (dose de-escalation), *n* = 8 0.5 mg/kg (initial dose), *n* = 14 3 mg/kg (dose escalation not realized due to toxicity) first application 21 mo. after allo-HCT (median)	with 1 mg/kg nivolumab CR/PR: 17/33% with 0.5 mg/kg nivolumab ORR/CR/PR/SD/PD 16/8/8/47/37% 6 mo. PFS/OS: 39/61%	with 1 mg/kg nivolumab sepsis/fatal ARDS (*n* = 1) fatal APS (*n* = 1) pneumonitis (III, *n* = 1) transaminitis (III, *n* = 1) bilirubinemia (III, *n* = 1)	with 1 mg/kg nivolumab aGvHD liver, gut (III, *n* = 2) with 0.5 mg/kg nivolumab new onset/worsening of GvHD (*n* = 10) aGvHD (N/A, *n* = 1) cGvHD (N/A, *n* = 7) aGvHD+ cGvHD (N/A, *n* = 2) time of GvHD onset after CI N/A GvHD related deaths N/A	[68]
10	r/r lymphoma	phase II multi-center study FL, *n* = 2; MCL, *n* = 3; THL, *n* = 1; DLBCL, *n* = 1; CLL, *n* = 2; ALCL, *n* = 1 no ongoing GvHD at study entry 44% had prior extensive cGvHD	lenalidomide (10 mg/day x 21d) + ipilimumab (3 mg/kg single-dose) 1st anti-CTLA-4 3d after completed 1st cycle of len repetition of len-cycle after 30d 2nd anti-CTLA-4 dose as before first application 29 mo. after allo- HCT (median)	ORR/CR/PR/SD 77/44/33/22% OS/PFS: N/A 4 mo RFS: 100% 12 mo RFS: 56%	hypothyrodism (II, *n* = 1)	cGvHD liver, mouth (N/A, *n* = 1) time of GvHD onset after CI N/A GvHD related deaths N/A	[72]

irAES: immune-related adverse events, a/cGvHD: acute/chronic Graft-versus-Host Disease, allo-HCT: allogeneic hematopoietic stem cell transplantation, r/r: relapsed and refractory, HL: Hodgkin’s lymphoma, FL: follicular lymphoma, mAb: monoclonal antibody, AML: acute myeloid leukemia, MDS: myelodysplastic syndrome, MF: myelofibrosis, NHL: Non-Hodgkin lymphoma, DLBCL: diffuse large B-cell lymphoma, HM: hematologic malignancy, CML: chronic myeloid leukemia, CLL: chronic lymphocytic leukemia, MPN: myeloid proliferative neoplasm, DLI: donor lymphocyte infusion, ITP: idiopathic thrombocytopenic purpura, MCL: mantle cell lymphoma, THL: triple hit lymphoma, ALCL: anaplastic large T-cell lymphoma, ARDS: acute respiratory deficiency syndrome, APS: antiphospholipid syndrome, ORR: overall remission rate, CR: complete remission, PR: partial remission, SD: stable disease, PD: progressive disease, OS: overall survival, PFS: progression free survival, RFS: remission free survival, N/A: not available, NOS: no otherwise specified.

**Table 2:** Overview of relevant studies targeting immune checkpoints after CAR T cell therapy.

n	Disease	Characteristics	Intervention	Response	irAEs (Grade)	CRS/ICANS (Grade)	Ref.
11	r/r B-NHL	retrospective single-center study DLBCL (Stage III-IV), *n* = 10 Burkitt’s lymphoma, *n* = 1	CD19 CAR + nivolumab (3 mg/kg single-dose) anti-PD-1 applied on d3 after CAR infusion	ORR/CR/PR/NR: 82/46/36/18% PFS: 6 (1–14 months)	no grade III-IV toxicity	CRS (I, *n* = 3; II, *n* = 6) ICANS (N/A, *n* = 1)	[103]
14	r/r B-ALL r/r B-NHL	retrospective single-center study B-ALL, *n* = 13 B-lymphoblastic lymphoma, *n* = 1	CD19 CAR + pembrolizumab (200 mg, q3w) CD19 CAR + nivolumab (3 mg/kg, q3w) anti-PD-1 application ≧ 14d after CAR T cell infusion, median time after CAR T cell infusion N/A	ORR/CR/PR/PD: 43/14/29/7% PFS: N/A	pancreatitis (N/A, *n* = 1) hypothyroidism (N/A, *n* = 1) urticaria (N/A, *n* = 1) arthropathy (N/A, *n* = 1) no grade V toxicities	CRS (N/A, *n* = 3)	[100]
4	r/r DLBCL	phase 1b prospective multi-center study (PORTIA) DLBCL, *n* = 4	CD19 CAR + pembrolizumab (200 mg, q3w, 6 courses) first anti-PD-1 application on d15 after CAR T cell infusion	N/A	none	CRS (N/A, *n* = 1)	[104]
12	r/r DLBCL	phase 1 prospective multi-center study (ZUMA-6) DLBCL, *n* = 12	CD19 CAR + atezolizumab (1200 mg, q3w, 4 courses) first anti-PD-L1 application on d21 (cohort 1, *n* = 3), d14 (cohort 2, *n* = 3), d1 (cohort 3, *n* = 6) after CAR infusion	ORR/CR/PR: 90/60/30% PFS: N/A	N/A	CRS (≥ III, *n* = 3) ICANS (≥ III, *n* = 6)	[105]
11	r/r DLBCL	phase 1/2 prospective multi-center study (ALEXANDER) DLBCL NOS, *n* = 4 DLBCL transformed from FL/MZL, *n* = 7	AUTO-3 CD19/CD22 CAR mono (*n* = 4) AUTO-3 CAR + pembrolizumab (200 mg, q3w) *n* = 7 first anti-PD-1 application on d14 Cohort 1: 50 × 10^6^ AUTO3, *n* = 7 Cohort 2: 150 × 10^6^ AUTO3, *n* = 4	Cohort 1: 50 × 10^6^ AUTO3 ORR/CR/PR: 57/29/28% PFS: N/A Cohort 2: 150 × 10^6^ AUTO3 N/A	N/A	CRS (I, *n* = 3) ICANS (III, *n* = 1)	[106]

CAR: chimeric antigen receptor, irAES: immune-related adverse events, CRS: cytokine release syndrome, ICANS: immune effector cell-associated neurotoxicity syndrome, r/r: relapsed and refractory, B-NHL: B-cell non-Hodgkin lymphoma, DLBCL: diffuse large B-cell lymphoma, B-ALL: B-cell acute lymphocytic leukemia, FL: follicular lymphoma, MZL: marginal zone B-cell lymphoma, CD: cluster of differentiation, ORR: overall remission rate, CR: complete remission, CRR: complete remission rate, PR: partial remission, PD: progressive disease, NR: no response, PFS: progression free survival, N/A: not available.

**Table 3:** Selected ongoing clinical trials targeting immune checkpoints after allogeneic hematopoietic cell transplantation or CAR T cell therapy.

Clinical Trial	Phase	Disease	Intervention	Sponsor
NCT02981914	I	r/r HL, B-NHL, AML, MDS after allo-HCT	pembrolizumab (q3w, 200 mg)	University of Chicago
NCT03286114	IB	r/r MDS, AML, ALL after allo-HCT	pembrolizumab (q3w, 200 mg)	University of Michigan Rogel Cancer Center
NCT04361058	I	r/r high risk AML, MDS after allo-HCT	nivolumab (q2w, 0.25 mg/kg, 4 courses)	SCRI Development Innovations, LLC
NCT02890329	I	r/r AML(+MRC), MDS after allo-HCT	decitabine + ipilimumab (q4w, dose N/A) priming: decitabine (d1-5 of 28 days) induction: decitabine (d1-5) + ipilimumab (d1); 4 courses maintenance: decitabine (d1-5) + ipilimumab (d1); 4 courses	National Cancer Institute (NCI)
NCT03588936	I	r/r AL, CL, MDS, lymphoma after allo-HCT	tocilizumab (8 mg/kg on day 0 and 29) + nivolumab (q2w, 0.25 or 0.5 mg/kg on day 1; up to 4 courses)	Medical College of Wisconsin
NCT03146468	II	r/r hematologic disease after allo-HCT	nivolumab (q2w, 3 mg/kg)	Melbourne Health
NCT01822509	I/IB	r/r AML, MDS, MPN, ALL, CLL, CML, (N)HL, MM after allo-HCT	nivolumab or ipilimumab induction: nivolumab (q2w, dose N/A, 8 courses) or ipilimumab (q3w, dose N/A; 4 courses) maintenance: nivolumab (q2w, dose N/A, up to a total of 60 weeks) or ipilimumab (q12w, dose N/A; 4 courses)	National Cancer Institute (NCI)
NCT03600155	IB	r/r high risk AML, MDS after allo-HCT	nivolumab or ipilimumab or nivolumab + ipilimumab Arm A: nivolumab (q4w, d1+15, dose N/A 6 courses) ≥ six weeks post allo-HCT Arm B: ipilimumab (q3w, d1, dose N/A, 6 courses) ≥ six weeks post allo-HCT Arm C; nivolumab (q6w, d1,14,28, dose N/A 6 courses) + ipilimumab (q6w, d1, dose N/A, 6 courses) ≥ six weeks post allo-HCT	M.D. Anderson Cancer Center
NCT00586391	I	B-NHL, CLL, ALL	CD19CAR-28-zeta T cells Dose Level 1: 2 × 10^7^ T cells/m^2^ Dose Level 2: 1 × 10^8^ T cells/m^2^ Dose Level 3: 2 × 10^8^ T cells/m^2^ ± ipilimumab (once in week 2 after CAR infusion, dose N/A, only in patients with low/intermediate grade leukemia/ lymphoma)	Baylor College of Medicine
NCT03630159	IB	r/r DLBCL	tisagenlecleucel + pembrolizumab timing and dose N/A	Novartis Pharmaceuticals
NCT03630159	I/II	r/r DLBCL	axicabtagene ciloleucel + atezolizumab timing and dose N/A	Kite, A Gilead Company
NCT03287817	I/II	r/r DLBCL	AUTO-3 (50 × 10^6^ to 900 × 10^6^ CD19/CD22 CAR T cells) ± pembrolizumab timing and dose N/A	Autolus Limited
NCT04134325	I	r/r HL after CAR T cell therapy	pembrolizumab (q3w, 200 mg) or nivolumab (q2w, 240 mg or q4w, 480 mg)	UNC Lineberger Comprehensive Cancer Center
NCT02650999	I/II	r/r DLBCL, FL, MCL after CAR T cell therapy	pembrolizumab (timing N/A, 200 mg)	Abramson Cancer Center of the University of Pennsylvania
NCT04205409	II	r/r CLL, DLBCL, FL, MZL, NHL, MM after CAR T cell therapy	nivolumab (q4w, dose N/A)	University of Washington
NCT04337606	I/II	r/r NHL after CAR T cell therapy	cohort 1: chidamide (q3w, 10 mg on d1-5 and 20 mg on d8,11,15,18) + decitabine (q3w, 10 mg on d1-5) cohort 2: decitabine (q3w, 10 mg on d1-5) + camrelizumab (q3w, 200 mg on d6)	Chinese PLA General Hospital

CAR: chimeric antigen receptor, allo-HCT: allogeneic hematopoietic stem cell transplantation, r/r: relapsed and refractory, HL: Hodgkin’s lymphoma, B-NHL: B-cell non-Hodgkin lymphoma, AML: acute myeloid leukemia, MDS: myeloid dysplastic syndrome, ALL: acute lymphocytic leukemia, MRC: myelodysplasia-related changes, N/A: not available, AL: acute leukemia, CL: chronic leukemia, MPN: myeloproliferative neoplasia, CLL: chronic lymphocytic leukemia, CML: chronic myeloid leukemia, NHL: Non-Hodgkin-lymphoma, MM: multiple myeloma, DLBCL: diffuse large B-cell lymphoma, FL: follicular lymphoma, MZL: marginal zone B-cell lymphoma.

## Abbreviations

aGvHD	acute Graft-versus-Host Disease
allo-HCT	allogeneic hematopoietic stem cell transplantation
ALL	acute lymphocytic leukemia
AML	acute myeloid leukemia
CAIX	carbonic anhydrase IX
CAR	chimeric antigen receptor
CD	cluster of differentiation
cGvHD	chronic Graft-versus-Host Disease
CI	checkpoint inhibition/inhibitor
CR	complete remission
CRS	cytokine release syndrome
CRISPR/Cas9	clustered regularly interspaced short palindromic repeats associated with Cas9 endonuclease
CTLA-4	cytotoxic T-lymphocyte-associated protein-4
DLBCL	diffuse large B-cell lymphoma
DLI	donor lymphocyte infusion
EGFR	epidermal growth factor receptor
EMA	European Medicines Agency
FDA	Food and Drug Administration
FL	follicular lymphoma
FOXP3 GD2	Forkhead-box-protein P3 disialoganglioside
GvHD	Graft-versus-Host Disease
GvL	Graft-versus-leukemia
GvT	Graft-versus-tumor
HD	Hodgkin’s disease
(c)HL	(classical) Hodgkin lymphoma
ICANS	immune cell associated neurotoxicity syndrome
ICOS	inducible T cell costimulator
irAEs	immune-related adverse events
KLRG-1	killer cell lectin-like receptor subfamily member 1
LAG-3	lymphocyte-activation gene 3
mAbs	monoclonal antibody
MDS	myelodysplastic syndrome
MGMT	0-6-Methylguanine DNA-methyltransferase
MiHA	minor histocompatibility antigen
MF	myelofibrosis
NHL	Non-Hodgkin-Lymphoma
ORR	overall response rate
OS	overall survival
PD-1	programmed death-1
PD-L1	programmed death- ligand 1
PFS	progression free survival
PMBCL	primary mediastinal B cell lymphoma
PR	partial remission
RNA	ribonucleotide acid
scFv	single chain variable fragment
SD	stable disease
TIGIT	T cell immunoglobulin and ITIM domains
TIM-3	T-cell immunoglobulin mucin-3
